# Epigenome-wide and transcriptome-wide analyses reveal gestational diabetes is associated with alterations in the human leukocyte antigen complex

**DOI:** 10.1186/s13148-015-0116-y

**Published:** 2015-08-05

**Authors:** Alexandra M. Binder, Jessica LaRocca, Corina Lesseur, Carmen J. Marsit, Karin B. Michels

**Affiliations:** Department of Epidemiology, Harvard School of Public Health, Boston, MA 02115 USA; Harvard University Center for the Environment, Harvard University, Cambridge, MA 02138 USA; Obstetrics and Gynecology Epidemiology Center, Department of Obstetrics, Gynecology, and Reproductive Biology, Brigham and Women’s Hospital, Harvard Medical School, 221 Longwood Ave., Boston, MA 02115 USA; Department of Pharmacology and Toxicology, and Section of Biostatistics and Epidemiology, Department of Community and Family Medicine, Geisel School of Medicine at Dartmouth, Hanover, NH 03755 USA

**Keywords:** Gestational diabetes, Methylome, Transcriptome, Human leukocyte antigen

## Abstract

**Background:**

Gestational diabetes mellitus (GDM) affects approximately 10 % of pregnancies in the United States and increases the risk of adverse health outcomes in the offspring. These adult disease propensities may be set by anatomical and molecular alterations in the placenta associated with GDM.

**Results:**

To assess the mechanistic aspects of fetal programming, we measured genome-wide methylation (Infinium HumanMethylation450 BeadChips) and expression (Affymetrix transcriptome microarrays) in placental tissue of 41 GDM cases and 41 matched pregnancies without maternal complications from the Harvard Epigenetic Birth Cohort. Specific transcriptional and epigenetic perturbations associated with GDM status included alterations in the major histocompatibility complex (MHC) region, which were validated in an independent cohort, the Rhode Island Child Health Study. Gene ontology enrichment among gene regulation influenced by GDM revealed an over-representation of immune response pathways among differential expression, reflecting these coordinated changes in the MHC region. This differential methylation and expression may be capturing shifts in cellular composition, reflecting physiological changes in the placenta associated with GDM.

**Conclusions:**

Our study represents the largest investigation of transcriptomic and methylomic differences associated with GDM, providing comprehensive insight into how GDM shapes the intrauterine environment, which may have implications for fetal (re)programming.

**Electronic supplementary material:**

The online version of this article (doi:10.1186/s13148-015-0116-y) contains supplementary material, which is available to authorized users.

## Background

Gestational diabetes mellitus (GDM) is the most common hyperglycemic disorder in pregnancy. Characterized by glucose intolerance that becomes clinically apparent near the end of the second trimester, the prevalence of GDM has doubled over the past 20 years, affecting approximately 10 % of pregnancies in the United States [1, 2]. In addition to shared clinical features, women with GDM have a substantially elevated risk of subsequent type 2 diabetes [3, 4], particularly in the presence of obesity [4, 5]. Exposure to GDM in utero may also adversely impact the health of the offspring, increasing the risk of macrosomia, and conferring a predisposition for obesity, metabolic syndrome, cardiovascular complications, and diabetes [1, 6]. While the clinical manifestations and potential implications of GDM for the mother and fetus have been well characterized, the molecular basis of GDM pathogenesis is largely unknown.

As the primary interface of nutrient transfer between mother and fetus, altered placental physiology has been suspected to be a key component of GDM pathogenesis and its impact on disease susceptibility in the offspring. Anatomical and molecular alterations in the placenta associated with GDM include significantly lower fetal-to-placental weight ratios [7], aberrant vascularization [8], and oxidative stress, potentially due to enhanced free radical production and/or defects in the antioxidant defenses [9]. Inadequate response to oxidative stress may contribute to enhanced inflammatory conditions, exacerbated by obesity [10]. Since fetal gluconeogenesis is minimal [11], the majority of the glucose essential to fetal growth and metabolism is transferred from the maternal circulation via the placenta. Transplacental glucose flux follows this maternal-to-fetal concentration gradient by facilitated diffusion, which can be characterized as flow-limited even at pathological glucose concentrations [12, 13]. Consequently, GDM exposes the fetus to abnormally high glucose levels, resulting in fetal hyperinsulinemia [14] and an increase in fetal fat mass [15]. GDM may additionally affect amino acid transport [16] and lipid concentrations, contributing to accelerated low-density lipoprotein oxidation [17].

The impact of this adverse intrauterine environment is in line with an abundance of epidemiologic evidence purporting the Developmental Origins of Health and Disease (DOHaD) [18, 19]. Due to their plasticity, epigenetic modifications provide one mechanism facilitating developmental adaptation to these conditions [20]. GDM has previously been associated with changes in placental gene expression across the genome [21–24], potentially reflecting changes in DNA methylation [25, 26]. In this study, we sought to understand the impact of GDM on the regulation of the placental transcriptome on the maternal (decidua basalis) side and how this is associated with alterations in the epigenome. This study represents the largest and most comprehensive integration of multi-omic data in this context, facilitating the identification of complex regulatory changes associated with GDM that may impact fetal (re)programming.

## Results and discussion

Genome-wide methylation and expression was assessed in the placentas (maternal-side) of 41 clinically confirmed cases of GDM and 41 matched pregnancies without maternal complications from the Harvard Epigenetic Birth Cohort (HEBC) at Brigham and Women’s Hospital, Boston, MA, USA. Samples were matched based on maternal age, pre-pregnancy BMI, method of conception, ethnicity, smoking status, and infant sex. Approximately 50 % of these women were normal weight (18.5 ≤ BMI < 25) prior to pregnancy. Aside from two previously underweight mothers, the remaining women were either overweight (20 %; 25 ≤ BMI < 30) or obese (29 %; 30 ≤ BMI). Birth weight and gestational age were not associated with GDM in this study population (Additional file 1: Table S1). Additional characteristics of the population are presented in Additional file 1: Table S1.

Among these 82 placenta samples, we assessed methylation changes across the genome associated with GDM using the 450K Infinium Methylation BeadChip. To identify site-specific differences, methylation level at each locus was modeled as a function of GDM, controlling for potential confounding variables (Additional file 1: Table S2; 20 most significant loci). Each model was adjusted for chart-abstracted maternal age, pre-pregnancy BMI, infant sex, maternal smoking, and independent surrogate variables associated with putative sources of internal bias measured with error, specifically self-reported maternal ethnicity and our indicators for batch (chip, row, and column). Due to their more intuitive biological interpretation, *β* values (range: 0–1; low to high methylation) were utilized to identify differentially methylated loci, using robust standard errors to account for possible heteroscedasticity associated with the impact of the methylation level on intrinsic variability. GDM was most significantly associated with a 0.00726-lower methylation level at one CpG locus within the intron of *CAPN1* (Additional file 1: Table S2). Given the limits of differential methylation detection above the technical variation of pyrosequencing, regions for validation were selected among the greatest absolute shifts in methylation associated with GDM, with a *p* value <0.001. In addition to these replication considerations, genomic context was factored into candidate selection, with priority given to regional changes detected via “bump hunting” (Additional file 1: Table S3).

**Fig. 1:**
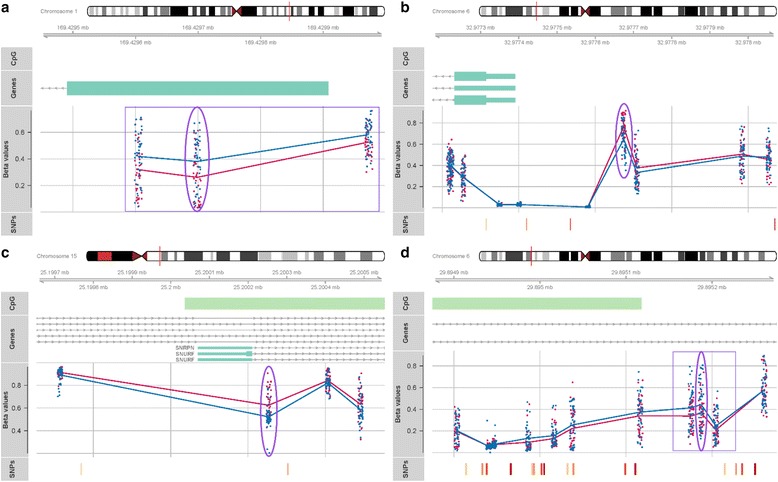
Regions selected for pyrovalidation based on observed association between GDM and methylation level on the microarray. *Purple ovals* highlight the CpG site driving the selection of each candidate region, with *purple boxes* indicating regional changes detected via bump hunting. Plots include site-specific methylation of GDM cases (*blue*) and matched controls (*pink*), and genomic context, including proximal CpG islands (*green*), HUGO genes (*teal*; smaller width corresponding to UTR), and SNPs colored according to heterozygosity (increasing from *yellow* to *red*). Regions include the following: **a** one within an enhancer and 5′UTR of *CCDC181*, **b** one 285-bp upstream of the transcription start site of *HLA-DOA*, **c** one associated with the promoter of *SNRPN/SNURF*, and **d** one within the introns of *HLA-H* and *HLA-J*

Based on this approach, we identified four candidate regions for verification in our samples and validation in an independent cohort (Fig. 1 and Table 1). These included one locus within an enhancer and 5′UTR of *CCDC181*, which was associated with a 0.137 (95 % CI: 0.068, 0.207) increase in placental methylation among GDM mothers in our adjusted model (cg25464921). A second locus, within the introns of *HLA-H* and *HLA-J*, also exhibited significantly higher methylation with GDM (cg23681866; 0.108 (95 % CI: 0.049, 0.168)). These two sites were additionally within the two largest differentially methylated regions associated with GDM (Additional file 1: Table S3). The remaining two loci chosen showed lower average methylation among the placentas of GDM mothers in our adjusted models. For the locus 285-bp upstream of the TSS of *HLA-DOA*, the average difference was 0.117 (cg08147094; 95 % CI: −0.177, −0.058), with a smaller difference of 0.089 (cg18506672; 95 % CI: −0.133, −0.044) at a locus associated with the promoter of *SNRPN/SNURF*. Validation of these associations was performed by pyrosequencing of placenta samples from GDM mothers and matched normal pregnancies selected from the Rhode Island Child Health Study (RICHS), consisting of mothers recruited following delivery at the Women and Infants Hospital of Rhode Island (Additional file 1: Table S4). Besides a regional change trending towards significance upstream of *HLA-DOA*, none of the associations estimated on the array replicated in the independent cohort (Table 1). In contrast, pyrosequencing of these regions in the HEBC identified significant differential methylation of similar magnitude to the changes estimated on the methylation array proximal to our four candidates and among surrounding loci (with a trend towards significance for the locus within *HLA-H/HLA-J*), thus verifying the array findings. A failure to validate may reflect a difference in the distribution of effect modifiers between the two study populations. Compared to the mothers from the HEBC, the RICHS mothers were younger, with a higher pre-pregnancy BMI, and a greater proportion self-reporting white ethnicity (Additional file 2: Figure S1). Accordingly, we investigated modification of the association between methylation and GDM by maternal age and BMI, dichotomized by the median of each in HEBC, as well as self-reported ethnicity (Additional file 1: Table S5). Among the RICHS placenta samples, we identified significant effect modification of the association between GDM and methylation in the 5′UTR of *CCDC181* by maternal age (*p* value for interaction *p* = 0.0122). Similar to the change observed in the HEBC cohort, GDM was associated with a trend towards higher methylation in older mothers, but among the younger mothers in RICHS, placentas had significantly lower average methylation at this context (−5.807 % (95 % CI: −10.890 and −0.724)), respectively. Within the region upstream of *HLA-DOA*, the impact of GDM differed by maternal pre-pregnancy BMI category in the validation cohort (*p* value for interaction *p* = 0.0387); in the higher BMI category, GDM was associated with lower methylation (−5.123 % (95 % CI: −8.825, −1.421)), as observed in HEBC. Significantly lower methylation of this *HLA-DOA*-associated region was also identified in the maternal blood of the HEBC samples among the women with a higher pre-pregnancy BMI (*p* value for interaction *p* = 0.0342; −6.039 % (95 % CI: −11.721, −0.357), Additional file 1: Tables S6–S7).

**Table 1:** Associations between GDM and methylation of candidate regions assayed by pyrosequencing in validation cohort (RICHS) and verification set (HEBC)

A. Locus closest to candidate on microarray^a^					
			Association with GDM [coef (95 % CI)]		
	Gene in proximity	CpG ID	450K array (beta)	Validation set (%)	Verification set (%)
	*HLA-DOA*	cg08147094	−0.12 (−0.18, −0.06)	−3.24 (−7.93, 1.44)	−13.95* (−20.50, −7.40)
	*HLA-H/HLA-J*	cg23681866	0.11 (0.05, 0.17)	−1.96 (−7.46, 3.54)	6.21 (−0.18, 12.59)
	*SNRPN/SNURF*	cg18506672	−0.09 (−0.13, −0.04)	−0.49 (−2.41, 1.43)	−7.24* (−11.44, −3.04)
	*CCDC181*	cg25464921	0.14 (0.07, 0.21)	−3.28 (−7.90, 1.34)	9.02* (3.53, 14.52)
B. Regional change within pyrosequenced region^b^					
			Association with GDM [coef (95 % CI)]		
	Gene in proximity	Size of region (Candidate)	450K array (beta)	Validation set (%)	Verification set (%)
	*HLA-DOA*	2 (CpG 1)	–	−3.01 (−6.17, 0.16)	−9.23* (−13.67, −4.79)
	*HLA-H/HLA-J*	3 (CpG 2)	–	−1.56 (−6.12, 3.01)	4.82 (−0.78, 10.42)
	*SNRPN/SNURF*	2 (CpG 1)	–	−0.44 (−4.70, 3.82)	−4.70 (−10.63, 1.23)
	*CCDC181*	8 (CpG 6)	–	−2.42 (−6.89, 2.04)	9.34* (3.41, 15.27)

^a^Association between GDM and methylation of CpG site assayed by pyrosequencing in closest proximity to the site chosen for validation based on methylation array data. The candidate locus in each region has the strongest association with GDM in our adjusted models. Linear model adjusted for maternal age (years), pre-pregnancy BMI (kg/m^2^), infant sex, maternal smoking (yes/no), and self-reported ethnicity. Positive values indicate an increase in methylation with GDM

^b^Change in methylation associated with GDM across pyrosequenced loci modeled using linear mixed models with a random intercept for sample, adjusting for the same covariates. Positive values indicate an increase in methylation with GDM

*p < 0.05

**Fig. 2:**
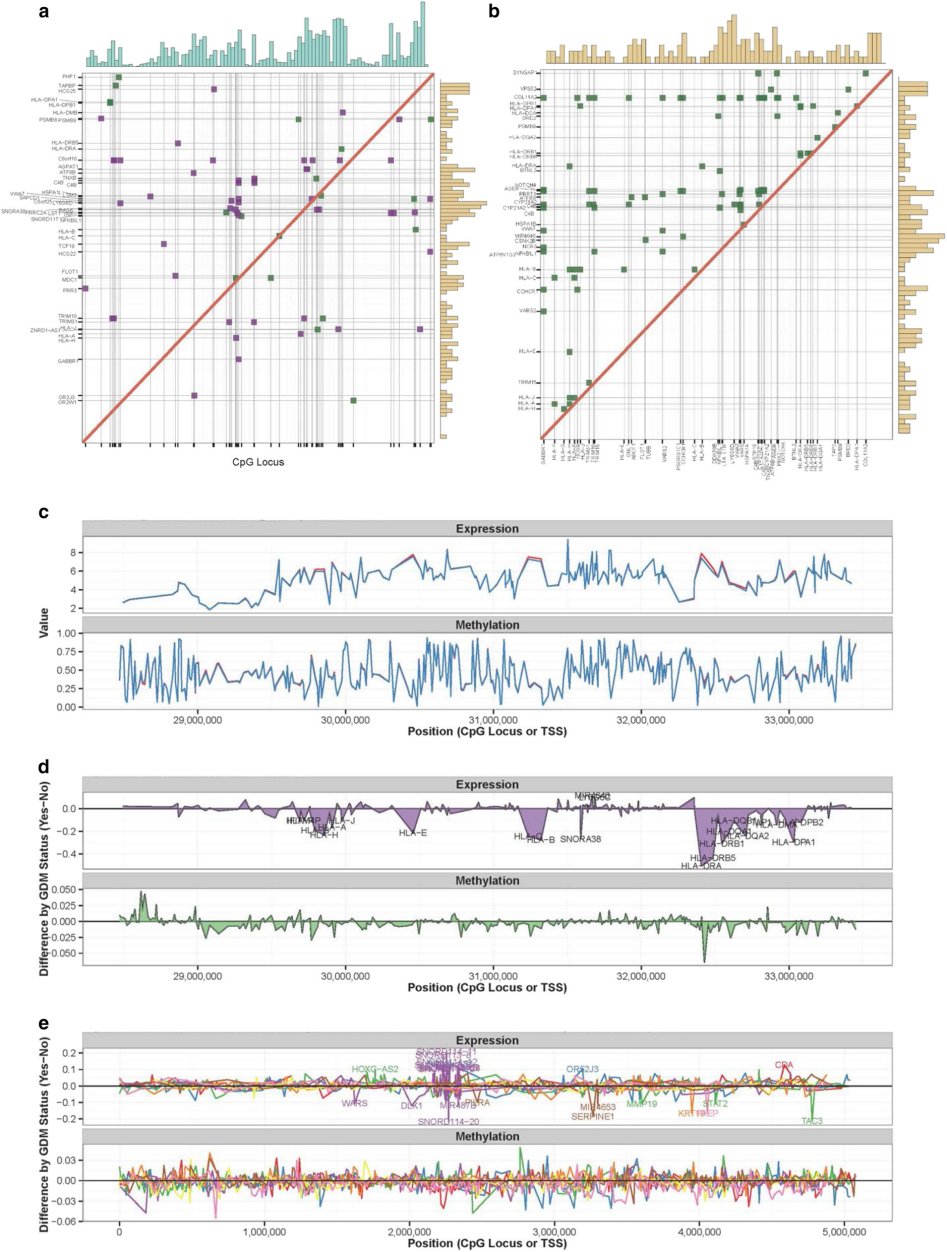
Coordinated regulation of expression and methylation in MHC region (chr6: 28477797–33448354). **a** Mapping the 72 regionally significant (*q* value <0.05) Spearman correlations between site-specific methylation and gene-level expression and **b** the 100 strongest, regionally significant (*q* value <0.05), pairwise Spearman correlations between gene expression levels. Mapped according to position on chromosome; *green* = positive correlation; *purple* = negative correlation; *tan histogram* indicates density of genes in region; *blue histogram* indicated density of CpG loci in region. **c** Plotting mean methylation and expression level for GDM cases (*blue*) and matched controls (*red*) and **d** the difference in mean methylation and expression between these groups across the MHC region. **e** Plotting difference in mean methylation and expression between GDM cases and controls across eight random regions to inform appraisal of coordinated regulation across MHC region. The eight regions included the following: chr1:16318931–21396338 (*red*), chr11:1868700–6913644 (*blue*), chr12:52616097–57666521 (*green*), chr14:98919898–103804291 (*purple*), chr17:35656660–40673466 (*orange*), chr5:174771462–179709813 (*yellow*), chr7:97501417–102576358 (*brown*), and chr9:134394271–139329735 (*pink*). Methylation level was collapsed across loci within 5 kb, restricting the maximum cluster width to 10 kb. Expression level was plotted at the transcription start site; for the plots of differential expression, *gene labels* were added for differences >0.10

In separate experiments, we assessed differential expression associated with GDM by interrogating expression levels across the genome in a subsample of 55 placentas assessed for genome-wide methylation changes using the Affymetrix Human Transcriptome Array 2.0. Similar statistical models were utilized to estimate associations between expression levels and GDM, adjusting for the same covariates used in our methylation models and our data-driven estimated surrogates for batch and ethnicity (Additional file 1: Table S8; top 20 unique coding genes, sorted by significance). Several of the most significant changes in coding genes were within the extremely polymorphic major histocompatibility complex (MHC) region on chromosome 6, represented by multiple haplotypes on the array. Playing a critical role in the immune response, this complex encodes genes involved in antigen presentation to T cells. Polymorphisms in the region have been previously implicated in genetic susceptibility to type 1 diabetes. GDM was associated with decreased expression of both MHC class I (e.g., *HLA-A*, *HLA-B*, and *HLA-C*) and MHC class II genes (e.g., *HLA-*DQA2) (Additional file 1: Table S8; Additional file 2: Figure S2). Given the observed differences in methylation associated with GDM in this same region, we investigated the correlation structure between methylation and gene expression in this region, as well as the correlation in expression among these genes (Fig. 2a, b). The verified locus upstream of *HLA-DOA* showed a weak positive correlation with *HLA-DOA* (*ρ* = 0.29), and a moderate positive correlation with several other MHC genes (*ρ* = 0.27–0.475), whereas the CpG locus within the intron of *HLA-H/HLA-J* was not correlated with any MHC genes (Additional file 1: Table S9). However, none of these comparisons reached regional significance (Additional file 1: Table S9). GDM was a stronger predictor of regional variation in expression across the MHC region than methylation (Fig. 2; Additional file 2: Figure S2). When averaged across large blocks of CpG loci in this region, GDM was not associated with shifts in methylation level, suggesting more site-specific impacts. In contrast, the enrichment for coordinated down-regulation of expression by GDM status was unique to the MHC region. Generally, coordinated changes across this region did not appear to be dependent on direct proximity, suggesting higher order regulation of these processes (Fig. 2). Three of the most significant associations with GDM in our adjusted models were verified by real-time PCR (qRT-PCR), chosen based on consistent results between the gene-level association with GDM and the exon-level associations for which primers could be designed. A similar trend towards decreased expression of *HLA-C* with GDM was observed, but the associations with *HLA-B* and *GPR174* were not replicated using a distinct technology (Additional file 1: Table S10). Validation of these genes by qRT-PCR was also attempted in the RICHS, but the results were not significant (Additional file 1: Table S10).

**Fig. 3:**
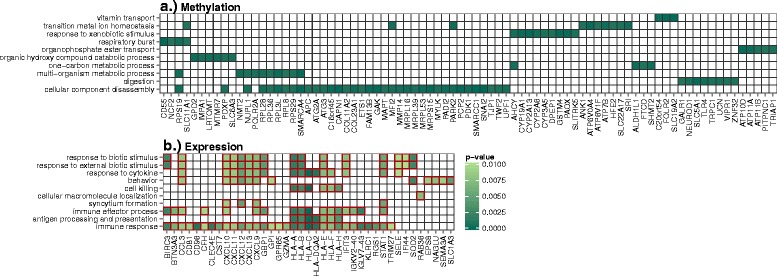
Biological process gene ontology (GO) enrichment among changes in **a** methylation and **b** expression associated with GDM. Plotting the 10 most significant biological processes after correcting for multiple testing (FDR). *Green boxes* indicate the genes in the significant subset driving the enrichment for each process; the color *green* corresponds to the significance of the association with GDM for each gene based on proximal methylation or expression; *red boxes* indicate ontologies with *q* value <0.1. All biological processes with *p* < 0.05 included in Additional file 1: Tables S11 and S12

Pathway-level variation associated with GDM was assessed by gene ontology (GO) enrichment among genes associated with methylation and expression changes. Compared to expression variation, methylation profiles are more stable and may be more strongly associated with early developmental patterns. Conversely, differential expression may be more reflective of maternal-fetal interface during later development. Therefore, each form of genome-wide data may be reflecting unique and meaningful molecular variation that may impact future growth patterns. Biological process GO enrichment was assessed among the genes in proximity to the 648 CpG loci associated with GDM in our adjusted models at *α* level =0.001. The most significantly enriched processes among methylation changes were associated with cellular metabolism and response to external stimuli. However, this enrichment was not significant after correcting for multiple testing (Additional file 1: Table S11; Fig. 3). Using a less stringent cutoff to identify ontologies enriched among differential expression patterns, the 171 genes associated with GDM in our adjusted expression models at *α* level =0.01 were strongly enriched for the immune response (Additional file 1: Table S12; Fig. 3). This enrichment reflected the strong association between GDM and expression in the MHC region (Fig. 3).

## Conclusions

This study represents the largest investigation of genome-wide variation in placental transcriptional regulation associated with GDM. Prior studies have assessed the impact of GDM on genome-wide expression patterns in the placenta, in both the fetal and maternal side, with numbers of diagnosed cases ranging from 2 to 19 [21–24]. While the genes with the largest change in expression associated with GDM varied between study populations, a majority of these studies identified enrichment for inflammatory pathways among observed differential expression. In one smaller study assessing variation across the fetal-side placental methylome, a distinct enrichment for metabolic disease pathways was observed [25]. Mirroring one of our findings, this prior study found GDM was strongly associated with a change in methylation in proximity to VIPR1, a gene suggested to play a role in controlling inflammation. Another study of 27 GDM-exposed South Asian mothers did not identify fetal-side methylation shifts that overlapped with either our study or the findings of Ruchet et al., suggesting possible race/ethnicity-specific changes [26]. Both prior studies of placenta methylation additionally investigated variation in cord blood methylation and found minimal overlap between site-specific shifts [25, 26]. Our study represents the first integration of genome-wide methylation and expression data to identify distinct and shared regulation. Through this multi-omic profiling, we identified markers of physiological changes in the placenta associated with intrauterine exposure to GDM. Given the influence of the placenta on fetal development, these regulatory patterns may contribute to the fetal (re)programming that has been associated with GDM exposure.

GDM shares several clinical features, lifestyle risk factors, and genetic susceptibility genes with type 2 diabetes [27, 28], including an increased incidence of chronic systemic inflammation [29–33]. In contrast to early insulin sensitivity, the later part of normal gestation is characterized by maternal hyperinsulinemia and insulin resistance, resulting in increased circulation of lipids and glucose to meet the energy requirements of fetal growth [34]. With amplified metabolic stress, such as with GDM and pre-existing obesity, peripheral insulin resistance is more pronounced and associated with increased concentrations of circulating fatty acids and lipids [15, 35]. The changes in insulin signaling may be induced by pro-inflammatory cytokines and adipokines secreted from both the adipose tissue and placenta of mothers with GDM [23, 36, 37], which have been previously implicated in insulin resistance among non-pregnant populations [38, 39]. Our findings further support the premise that a diabetic environment in utero instigates the overexpression of inflammation-related genes in the placenta and suggest the increased release of inflammatory molecules. These shifts in expression may be more generally reflective of the shifts in placenta cell composition associated with the systemic inflammation characteristic of GDM.

In addition to this general immunomodulation, we identified coordinated regulation in the major histocompatibility complex (MHC) region associated with GDM, suggesting a potential autoimmune component in pathogenesis that is more characteristic of type 1 diabetes. Contrary to the insulin resistance of type 2 diabetes, type 1 diabetes is characterized by the progressive destruction on pancreatic beta cells as a result of T-cell-mediated autoimmunity, leading to insulin deficiency and hyperglycemia. Polymorphisms within class II human leukocyte antigen (HLA) genes coded in the MHC region have been implicated in predispositions for type 1 diabetes [40–42]. These class II molecules play a role in the presentation of antigenic peptides to helper T cells, whereas class I molecules present to cytotoxic T cells. Compared to the number of studies focused on the clinical features shared with type 2 diabetes, there is a relative paucity of studies assessing the similarities between GDM and type 1 diabetes. Approximately 10 % of GDM is suspected to represent an autoimmune form, characterized by the presence of beta-call cell autoantibodies [43], an increased frequency of type-1-diabetes-related haplotypes in HLA class II genes [44, 45], and a higher risk of late-onset type 1 diabetes [46–48]. More generally, GDM has been associated with increased anti-HLA-class-II antibodies in maternal circulation, suggesting reduced tolerance towards alloantigens [49], which may play a role in GDM pathogenesis through inflammatory activation. Our findings highlight a significant role of the MHC region in the presentation of GDM, with a general down-regulation of HLA genes among GDM-exposed placentas. While GDM has not been associated with an increased risk of type 1 diabetes in the offspring, shifts in autoimmune cells in the placenta may impair the intrauterine environment, shaping fetal development. It is possible that the adverse changes associated with GDM may be exacerbated by a higher pre-pregnancy BMI or higher maternal age, as evidenced by our methylation stratification results. Decreased placental methylation upstream of *HLA-DOA* among GDM cases was observed in both the HEBC and RICHS cohorts, particularly among the women with a high pre-pregnancy BMI. In maternal blood samples, we saw a similar decrease in methylation in this region among these women. These results highlight the heterogeneity of GDM but also suggest that additional investigation into the regulation of the MHC region associated with GDM is warranted.

Differential methylation and expression associated with GDM likely also captures shifts in cellular composition, reflecting physiological changes in the placenta. GDM has previously been associated with gross anatomical changes, including increased placental weight, diameter, and thickness among poorly controlled diabetic women [8]. Our samples were excised from the maternal (decidua basalis) side, which primarily contains cytotrophoblasts, syncytiotrophoblasts, and extravillous trophoblasts. Previously observed histological changes associated with GDM include villous edema, fibrin deposits in the syncytiotrophoblast, and cytotrophoblast hyperplasia [8]. Changes in the regulation of immunomodulatory genes in the placenta identified in this and prior studies may reflect the infiltration of inflammatory cells into the placenta of diabetic mothers. Unfortunately, purified placenta cell types have not previously been analyzed on the Illumina Infinium array to estimate and adjust for the impact of cellular composition on our results [50]. However, these shifts in composition are potentially on the causal pathway. Removing this component of the variation in methylation and expression would have likely precluded identification of the autoimmune component that has not been thoroughly explored by prior studies. This is a biological variation of interest given its role in shaping the intrauterine environment.

Our study exhibits several strengths, including a large population size, the integration of genome-wide methylation and expression, the employment of technical verification, and the use of an independent cohort for validation. Beyond the general assessment of reproducibility, validation in an independent cohort helped to identify maternal characteristics that modified observed epigenetic changes, further elucidating condition heterogeneity. While our candidate methylation changes were verified in our study population by pyrosequencing, none of these shifts were significantly reproduced in our validation cohort. For two of these regions, a failure to validate reflected a difference in the distribution of measured effect modifiers between the two study populations. Additional effect modification by unmeasured factors, or additional unmeasured confounding, may account for the inability to validate certain regions. Due to our sample size, we could also not explore the possibility of more complex models of interaction that may influence the association. The failure to validate the expression microarray results by qRT-PCR results may partially reflect technical differences between the platforms. For instance, the qRT-PCR probes span larger lengths of the transcriptome than what is represented with the exon levels analyzed by the microarray. In addition, the greater temporal variability of expression may make these changes generally more difficult to validate. Future larger, more directed studies may be able to further refine the specific populations susceptible to molecular changes associated with GDM.

While methylation may be capturing regulatory patterns established early in gestation or earlier shifts in cellular composition, the identification of differential expression is restricted to genes expressed perinatally. By interrogating the methylome and transcriptome, we are able to characterize the influence of GDM on placental gene regulation across gestation. This integration provides comprehensive insight into the molecular basis of GDM pathogenesis with possible implications for fetal (re)programming.

## Availability of supporting data

The data sets supporting the results of this article have been deposited in NCBI’s Gene Expression Omnibus and are accessible through GEO SuperSeries accession number GSE70494 (http://www.ncbi.nlm.nih.gov/geo/query/acc.cgi?acc=GSE70494).

## Methods

### Study population

Our study population consisted of women enrolled in the Harvard Epigenetic Birth Cohort (HEBC) at the Brigham and Women’s Hospital (BWH) in Boston, MA. The HEBC was initiated to study prenatal determinants of epigenetic marks in cord blood and placenta. Data and biospecimens for the HEBC were collected from June 2007 to June 2009 and include 1941 mother-child dyads [51]. Mother-infant dyads for the proposed study have been selected as follows: “case” placenta samples were selected among mothers with a clinical diagnosis of gestational diabetes, excluding women with pre-existing hypertension or pre-existing diabetes mellitus. “Controls” were identified among samples with no gestational diabetes, no pregnancy-induced hypertension, no preeclampsia, and no previous hypertension or diabetes. Each case was individually matched to a control with a maternal age within 5 years of the case, with the same method of conception, ethnicity, smoking status, and infant sex, and with the closest pre-pregnancy BMI among mothers in the HEBC. Matching criteria was relaxed when the difference in pre-pregnancy BMI between the case and closest control match was >5 kg/m^2^. Characteristics for the case and control samples are summarized in Additional file 1: Table S1; continuous variables are summarized by the mean (SD), and categorical variables are reported as counts (%).

### Ethics statement

The study protocol was approved by the Institutional Review Board of the Brigham and Women’s Hospital. Completing the pregnancy questionnaire was considered implied consent.

### Sample preparation

Placenta and maternal blood samples were collected immediately after delivery. Tissue samples collected for DNA extraction were snap-frozen and stored in liquid nitrogen, with samples for RNA extraction stored in RNAlater (Ambion, Carlsbad, CA) at −20 °C until further processing. All placenta samples used in this study were taken from the maternal (decidua basalis) side near the umbilical cord.

### DNA isolation

DNA was isolated from placenta tissue and maternal blood samples using the QIAmp DNA Mini Kit (Qiagen) according to the manufacturer’s instructions.

### Illumina 450K methylation microarrays

Genomic DNA was bisulfite treated using the Zymo EZ-96 DNA methylation kit (Zymo Research). For the assessment of genome-wide DNA methylation, the Illumina 450K Infinium Methylation BeadChip was used. The distribution of samples across chips was blinded by randomly sorting de-identified matched samples to reduce the likelihood of systematic technical bias. This array covers approximately 99 % of RefSeq genes, with approximately 17.2 probes per gene region, and 96 % of CpG islands, including the more tissue-specific CpG island shores and shelves [52]. The 450K methylation microarrays were performed at The University of Southern California’s (USC, CA, USA) USC Epigenome Center (http://epigenome.usc.edu/).

### Validation and verification of DNA methylation using pyrosequencing

Four differentially methylated regions (Additional file 1: Table S13) associated with GDM in our adjusted models were verified in the HEBC and validated in a separate birth cohort, RICHS. RICHS is an ongoing, population-based birth cohort established at Women and Infants’ Hospital of Rhode Island and independently funded (R01 MH094609) [53, 54]. A total of 500 ng of genomic DNA was bisulfite converted using the Zymo EZ-96 DNA methylation kit (Zymo Research).

For each region of interest, PCR was performed using a primer set without CG dinucleotides in their sequences specific for the converted DNA. These primers surrounded at least three CG dinucleotides and enabled us to amplify both methylated and unmethylated templates. Optimized primers were ordered from EpigenDx (EpigenDx, Hopkinton, MA; design described in Additional file 1: Table S13). Amplification products were sequenced on our PyroMark Q24 pyrosequencer (Qiagen). Another bisulfite conversion check was performed by adding cytosine site in the sequence to assess incomplete conversion of the DNA strands. Percent methylation was estimated by the proportion C/(C + T) at each CG site within the amplified region.

### RNA isolation

Using the mirVANA RNA Isolation Kit (Ambion Inc., Austin, TX), RNA was isolated according to the manufacturer’s protocol.

### Affymetrix gene expression arrays

Gene expression was assessed using the Human Transcriptome Array 2.0 (Affymetrix). The array is designed with a median of approximately 21 unique probes per transcript, enabling analysis of expression at the gene level and investigation of alternative splicing variants. The probes on the array cover a total of >30,000 coding transcripts, including alternative splice variants, and >11,000 lincRNA. Transcript coverage and gene count correspond to RefSeq from February, 2012. A total of 50 ng of high-quality RNA (260/280 = 1.8–2.0; RIN > 6.0) was used to generate sense-strand cDNA with the Ambion® WT Expression Kit (Life Technologies). The Affymetrix gene expression arrays were performed at the Center for Personalized Genetic Medicine, a research core facility of the Partners Health Care System in Cambridge, MA. Expression arrays were performed on 55 case-control samples plus quality controls.

### Real-time qRT-PCR

The expression levels of three genes that differed significantly among GDM cases and controls were chosen for verification in the HEBC and validation in RICHS. cDNA was synthesized using 500 ng of RNA with the High Capacity cDNA Reverse Transcription Kit (Applied Biosystems, Foster City, USA). Predesigned PrimeTime qPCR Assays from Integrated DNA Technologies (IDT) were used for *HLA-C*, *HLA-B*, and *GPR174*. The qRT-PCR reaction was performed with TaqMan Gene Expression Master Mix (Applied Biosystems) according to the manufacturer’s instructions on a Life Technologies 7900HT qPCR machine at the Harvard Medical School ICCB Screening Facility with reverse transcription controls. The qRT-PCR cycling conditions were as follows: 50 °C for 2 min, 95 °C for 10 min, 40 cycles of 95 °C for 15 s, and 60 °C for 60 s. All qRT-PCR data was normalized using the PrimeTime *GAPDH* Assay (IDT). Delta Ct (ΔCt) was defined as the expression difference between the target gene and GAPDH: ΔCt = Ct_Gene of Interest_ – Ct_GAPDH._ All samples were analyzed in triplicate. Additional file 1: Table S14 lists primer information.

### Statistical analysis

Methylation data was processed prior to analysis in accordance with best practices to reduce the influence of technical artifacts [55]. Color bias adjustment and quantile normalization (QN) was performed on signal intensities to decrease technical variation, specifically array position effects [56]. The fluorescence intensities from methylated (M) and unmethylated (U) alleles were then converted to methylation level, ranging from 0 to 1, given by *β* = M/(M + U + 100). Adjustment for probe-type bias was performed on the *β* values using beta-mixture quantile normalization (BMIQ) [57, 58]. We restricted further analysis to autosomal loci that did not have a SNP at the target locus or have a probe that cross-hybridized to the X chromosome, which could induce gender bias due to X inactivation [59]. Even after between-array normalization, batch effects may contribute bias in site-specific analyses [60]. Independent surrogate variable analysis (ISVA) was utilized to estimate this technical confounding based on our surrogates for batch (chip, chip row, chip column), as well as biological confounders assumed to be measured with error (self-reported ethnicity) [61]. Estimated independent surrogate variables (ISVs) associated with these confounders were incorporated into subsequent multivariable models. Site-specific methylation was modeled as a function of GDM status, adjusting for maternal age (years), pre-pregnancy BMI (kg/m^2^), infant sex, maternal smoking (yes/no), and our ISVs. To account for possible heteroscedasticity due to the intrinsic association between methylation level and variability, we used robust standard errors in Wald tests for our indicator of GDM status. Regional changes were identified using a “bump hunting” approach, using the T statistic from our adjusted models to identify differentially methylated regions among contiguous loci within 500 bp that exceed the 99th percentile of genome-wide changes, summarizing these regions based on the effect size [62]. Candidates were selected after removing probes with a SNP or non-specific binding to other autosomal sites, which, while potentially true signals, would increase the difficulty of primer design. Associations with GDM across pyrosequenced loci were modeled using linear mixed models with a random intercept for sample, adjusting for maternal age, pre-pregnancy BMI, infant sex, maternal smoking, and self-reported ethnicity. Site-specific analyses were also performed for the locus in closest proximity to the candidate on the array. Possible effect modification by maternal ethnicity (white/non-white), maternal age (dichotomized by median age in HEBC), and pre-pregnancy BMI (dichotomized by median BMI in HEBC) was assessed among the pyrosequenced regions by a Wald test of the interaction term.

CEL files containing the measured expression intensities were processed using the Affymetrix Expression Console Software (Affymetrix). Background correction was performed using the Robust Multichip Analysis (RMA) algorithm, to minimize the variance seen across arrays [63]. These probe values were then quantile normalized and summarized into one gene-level expression measure using median polish [63, 64]. ISVs significantly associated with our surrogates for batch (plate row, plate column) and maternal ethnicity in the expression matrix were incorporated into subsequent models. Associations between GDM and expression were then adjusted for the covariates included in our methylation models, using robust SEs in our Wald tests. For ease of interpretation, only associations with coding RefSeq genes were reported. To identify coordinated regulation between methylation and expression, as well as co-expression networks in the MHC region (chr6: 28477797–33448354), we estimated the Spearman correlation between each CpG within 10 kb of the MHC region and the expression of each gene in this region, as well as between the expression of each gene in this region. The significance of each pairwise comparison was estimated by 10,000 permutations, imputing 1e−6 for *p* = 0. We applied the Benjamini-Hochberg (false discovery rate) method to correct the two-sided *p* values for multiple testing across the MHC region.

Biological process gene ontology (GO) enrichment was assessed among changes in methylation and expression associated with GDM. We restricted analysis to ontologies associated with between 20 and 1500 genes and used the information content of these terms to identify potential redundancy. The Jiang and Conrath method was used to calculate the pairwise similarity between terms, using a scaled (0–1) cutoff of 0.7 to define a clustering of “highly similar terms.” GO analysis was then restricted to the GO associated with the largest number of genes among highly similar terms, resulting in a final subset of 402 biological processes. Enrichment of these terms was assessed among the genes in proximity to the 648 CpG loci associated with GDM in our adjusted models at *α* level =0.001. The non-random design of the methylation array precluded standard significance testing of this enrichment. Certain genes are represented by more probes on the array, potentially due to CpG island proximity or scientific interest, increasing the likelihood of detecting enrichment among terms associated with these genes. To account for this design bias, a resampling approach was used. For each GO analyzed, 648 CpG loci were randomly selected from the array and assigned to the closest gene. This random sampling was repeated 10,000 times to approximate the null odds ratio distribution of each term on the array and calculate the significance of observed methylation enrichment. Biological process enrichment among expression changes associated with GDM (*p* < 0.01; 171 genes) was assessed using hypergeometric tests. The significance of methylation and expression GO enrichment was then corrected for the false discovery rate, estimating associated *q* values [66].

## Additional files

Additional file 1: Table S1. Characteristics of placenta samples selected from the HEBC cohort that were assessed on the Illumina Infinium array. **Table S2.** Most significant changes in methylation associated with GDM in adjusted model. **Table S3.** Largest regional changes in methylation associated with GDM. **Table S4.** Characteristics of placenta samples selected from the RICHS cohort that were used to validate methylation changes observed in HEBC. **Table S5.** Assessing effect modification of the association between GDM and placenta methylation level estimated by pyrosequencing by maternal age, ethnicity, and pre-pregnancy BMI in our validation cohort (RICHS) and verification set (HEBC). **Table S6.** Among maternal blood HEBC samples, associations between GDM and methylation of candidate regions assayed by pyrosequencing. **Table S7.** Assessing effect modification of the association between GDM and maternal blood methylation level estimated by pyrosequencing by maternal age, ethnicity, and pre-pregnancy BMI in HEBC. **Table S8.** Most significant changes in gene-level expression associated with GDM in adjusted model, restricting to top 20 unique coding RefSeq genes. **Table S9.** Spearman correlation between methylation of loci chosen for validation and expression of genes in MHC region (chr6: 28477797–33448354), restricting to pairwise comparisons with *p* < 0.05. **Table S10.** Association between GDM and expression of genes estimated by qRT-PCR in adjusted models in our validation cohort (RICHS) and verification set (HEBC). **Table S11.** Biological pathway enrichment among genes in proximity to methylation changes associated with GDM. **Table S12.** Biological pathway enrichment among genes with differential expression associated with GDM. **Table S13.** Pyrosequencing assay information. **Table S14.** qRT-PCR primer information. Additional file 2: Figure S1. Distribution of maternal characteristics in HEBC and validation cohort (RICHS). **Figure S2.** Plotting the first two principle components for methylation and expression in the MHC region (chr6: 28477797–33448354) for GDM cases (blue) and matched controls (red). **Figure S3.** The subset of 20 genes driving the first two principle component loadings for expression (|rotation| > 0.1) in the MHC region (chr6: 28477797–33448354) for GDM cases (blue) and matched controls (red).
