# Supplementary material for: Epigenome-wide and transcriptome-wide analyses reveal gestational diabetes is associated with alterations in the human leukocyte antigen complex
Source: Clin Epigenetics. 2015 Aug 5;7(1):79. doi: 10.1186/s13148-015-0116-y (PMC4524439; doi:10.1186/s13148-015-0116-y)
Supplement: Additional file 1: Table S1. — Characteristics of placenta samples selected from the HEBC cohort that were assessed on the Illumina Infinium array. Table S2. Most significant changes in methylation associated with GDM in adjusted model. Table S3. Largest regional changes in methylation associated with GDM. Table S4. Characteristics of placenta samples selected from the RICHS cohort that were used to validate methylation changes observed in HEBC. Table S5. Assessing effect modification of the association between GDM and placenta methylation level estimated by pyrosequencing by maternal age, ethnicity, and pre-pregnancy BMI in our validation cohort (RICHS) and verification set (HEBC). Table S6. Among maternal blood HEBC samples, associations between GDM and methylation of candidate regions assayed by pyrosequencing. Table S7. Assessing effect modification of the association between GDM and maternal blood methylation level estimated by pyrosequencing by maternal age, ethnicity, and pre-pregnancy BMI in HEBC. Table S8. Most significant changes in gene-level expression associated with GDM in adjusted model, restricting to top 20 unique coding RefSeq genes. Table S9. Spearman correlation between methylation of loci chosen for validation and expression of genes in MHC region (chr6: 28477797–33448354), restricting to pairwise comparisons with p < 0.05. Table S10. Association between GDM and expression of genes estimated by qRT-PCR in adjusted models in our validation cohort (RICHS) and verification set (HEBC). Table S11. Biological pathway enrichment among genes in proximity to methylation changes associated with GDM. Table S12. Biological pathway enrichment among genes with differential expression associated with GDM. Table S13. Pyrosequencing assay information. Table S14. qRT-PCR primer information. [file 13148_2015_116_MOESM1_ESM.docx]

**Additional file 1**

**Table S1.** Characteristics of placenta samples selected from the HEBC cohort that were assessed on the Illumina Infinium Array. GDM “cases” were matched to a “control” pregnancy without maternal complications based on maternal age, method of conception, ethnicity, smoking status, infant sex, and pre-pregnancy BMI. Continuous variables are summarized by the mean (SD) and categorical variables are reported as counts (%).

| Characteristic | | | Cases (n=41) | Controls (n=41) | p-value |
| --- | --- | --- | --- | --- | --- |
| Pre-pregnancy BMI (kg/m^2^) | | | 26.653 (5.733) | 26.410 (5.422) | p= 0.7635 |
| Maternal Age (years) | | | 33.171 (4.652) | 33.487 (4.853) | p= 0.7635 |
| Gravidity | | |  |  | p= 0.3891 |
|  | | 1 | 8 (19.51%) | 4 (9.76%) |  |
|  | | 2 | 11 (26.83%) | 16 (39.02%) |  |
|  | | 3 | 13 (31.71%) | 10 (24.39%) |  |
|  | | >4 | 8 (19.51%) | 11 (26.83%) |  |
| Smoke during Pregnancy | | |  |  |  |
|  | No | | 38 (92.683%) | 38 (92.683%) | p=1 |
|  | Yes | | 3 (7.317%) | 3 (7.317%) |  |
| Infant Sex | | |  |  |  |
|  | Males | | 20 (48.78%) | 21 (48.78%) | p=1 |
|  | Females | | 21 (51.22%) | 20 (51.22%) |  |
| Ethnicity | | |  |  |  |
|  | Non-Hispanic White | | 23 (56.098%) | 23 (56.098%) | p=0.9437 |
|  | Hispanic or Latino | | 5 (12.195%) | 4 (9.756%) |  |
|  | Asian/Pacific Islander | | 7 (17.073%) | 6 (14.634%) |  |
|  | Black/African-American | | 6 (14.634%) | 8 (19.512%) |  |
| Conception | | |  |  |  |
|  | Spontaneous planned | | 24 (58.537%) | 24 (58.537%) | p=1 |
|  | Spontaneous unplanned | | 12 (29.268%) | 12 (29.268%) |  |
|  | Ovulation-induction drug | | 2 (4.878%) | 2 (4.878%) |  |
|  | IVF | | 3 (7.317%) | 3 (7.317%) |  |
| Gestational Age (weeks) | | | 39.077 (0.932) | 39.206 (1.047) | p= 0.5576 |
| Birth Weight (kg) | | | 3.542 (0.535) | 3.506 (0.502) | p= 0.7590 |

**Table S2.** Most significant changes in methylation associated with GDM in adjusted model.

| ILMNID | Coefficient† | P-value‡ | Chrom. | Position | Relation to Island | Regulatory Feature | Closest TSS (bp) | Gene Name |
| --- | --- | --- | --- | --- | --- | --- | --- | --- |
| cg01570480 | -0.0073 | 4.76E-09 | 11 | 64951916 | Island | Enhancer | 2613 | CAPN1 |
| cg16212074 | 0.0959 | 6.64E-07 | 19 | 4170115 | N Shelf |  | 12481 | SIRT6 |
| cg04920227 | 0.0053 | 2.37E-06 | 20 | 13202476 | Island |  | 59 | ISM1 |
| cg23163783 | 0.0035 | 2.76E-06 | 12 | 62586568 | S Shore | Unclassified | 52 | FAM19A2 |
| cg26206910 | 0.0047 | 3.14E-06 | 14 | 36277894 | Island | Promoter Associated | 538 | RALGAPA1 |
| cg03791150 | 0.0642 | 6.05E-06 | 3 | 42542232 | N Shore |  | -1871 | VIPR1 |
| cg12141457 | -0.0193 | 7.16E-06 | 10 | 126182012 |  |  | 31672 | LHPP |
| cg00837398 | 0.0179 | 8.31E-06 | 7 | 148958537 | N Shore | Promoter Associated | -724 | ZNF783 |
| cg01423426 | 0.0117 | 9.08E-06 | 8 | 119064312 |  | Enhancer | 36186 | AK025288 |
| cg05232889 | 0.0329 | 9.56E-06 | 7 | 114055419 |  |  | 368 | FOXP2 |
| cg24498760 | -0.0140 | 1.01E-05 | 8 | 49746867 | N Shore |  | 87132 | SNAI2 |
| cg18351406 | -0.0218 | 1.18E-05 | 4 | 77819688 | S Shore |  | -686 | SOWAHB |
| cg07113947 | 0.0051 | 1.21E-05 | 1 | 207494596 | Island | Promoter Associated | -220 | CD55 |
| cg22984439 | 0.0059 | 1.25E-05 | 3 | 47823638 | Island | Promoter Associated | -42 | SMARCC1 |
| cg08085639 | -0.0570 | 1.33E-05 | 19 | 1506135 | Island |  | 5000 | ADAMTSL5 |
| cg00114160 | 0.0600 | 1.39E-05 | 6 | 29430096 |  |  | 550 | OR2H1 |
| cg07304526 | 0.0250 | 1.48E-05 | 10 | 124221058 | Island | Cell type specific | 18 | HTRA1 |
| cg27035678 | 0.0047 | 1.49E-05 | 22 | 20861940 | Island | Promoter Associated | 36 | MED15 |
| cg25833003 | 0.0417 | 1.52E-05 | 10 | 101822013 | N Shelf |  | 19629 | CPN1 |
| cg11834681 | -0.0706 | 1.82E-05 | 19 | 41594746 | N Shore |  | 379 | CYP2A13 |

**†**Adjusting for chart-abstracted maternal age, pre-pregnancy BMI, infant sex, maternal smoking, and independent surrogate variables associated with putative sources of internal bias measured with error; specifically, self-reported maternal ethnicity and our indicators for batch (chip, row, and column). Positive values indicate an increase in methylation with GDM.

**‡** Beta-values were utilized to identify differentially methylated loci, using robust standard errors to account for possible heteroscedasticity

**Table S3.** Largest regional changes in methylation associated with GDM. Regional changes were identified using a “bump hunting” approach, using the T-statistic from our adjusted models to identify differentially methylated regions among contiguous loci within 500 bp that exceed the 99^th^ percentile of genome-wide changes, summarizing these regions based on the effect size (area: summation of coefficients from site-specific models); restricted to regions with at least three associated CpG loci.

| Chrom. | Start | End | Area | Size | Associated CpG Loci |
| --- | --- | --- | --- | --- | --- |
| chr1 | 169429604 | 169429972 | 0.31773 | 3 | cg09671955;cg16136262;cg25464921 |
| chr6 | 29895175 | 29895204 | 0.281098 | 3 | cg15411272;cg23681866;cg26121931 |
| chr8 | 25905636 | 25905811 | 0.270074 | 3 | cg02745262;cg06455149;cg24923694 |
| chr2 | 740151 | 740718 | 0.186238 | 4 | cg02758766;cg04344532;cg10414026;cg22595526 |
| chr14 | 102027514 | 102027734 | 0.182816 | 4 | cg01664864;cg02287710;cg15238382;cg15341124 |
| chr12 | 4405469 | 4405584 | 0.173137 | 3 | cg01264332;cg02401639;cg07181862 |
| chr16 | 17562960 | 17563300 | 0.151477 | 3 | cg01724917;cg04359840;cg06321596 |
| chr5 | 170735186 | 170735347 | 0.149915 | 3 | cg05787556;cg18336674;cg26517171 |
| chr1 | 42384365 | 42384474 | 0.145247 | 3 | cg03884592;cg25607920;cg26038582 |
| chr3 | 192445533 | 192445582 | 0.137811 | 3 | cg06374165;cg06804312;cg22069098 |
| chr3 | 183817230 | 183817853 | 0.135597 | 3 | cg05451388;cg11158819;cg24875857 |
| chr5 | 140729605 | 140729614 | 0.107951 | 3 | cg03447435;cg17557357;cg25592513 |
| chr6 | 30881560 | 30881571 | 0.107207 | 3 | cg05267955;cg15080939;cg18261909 |
| chr19 | 1371027 | 1371281 | 0.072728 | 3 | cg14062238;cg19037241;cg25566714 |

**Table S4.** Characteristics of placenta samples selected from the RICHS cohort that were used to validate methylation changes observed in HEBC. GDM “cases” were matched to a “control” pregnancy without maternal complications based on maternal age, method of conception, ethnicity, smoking status, infant sex, and pregnancy BMI. Continuous variables are summarized by the mean (SD) and categorical variables are reported as counts (%).

| Characteristic | | Cases (n=51) | Controls (n=51) | p-value |
| --- | --- | --- | --- | --- |
| Pre-pregnancy BMI | | 28.32 (5.76) | 28.2 (5.67) | p=0.914 |
| Maternal Age | | 30.69 (4.61) | 30.75 (4.55) | p=0.948 |
| Gravidity | |  |  | p=0.294 |
|  | 1 | 6 (11.76%) | 10 (19.61%) |  |
|  | 2 | 16 (31.37%) | 20 (39.22%) |  |
|  | >3 | 29 (56.86%) | 21 (41.18%) |  |
| Maternal Smoking | |  |  | p=1 |
|  | No | 47 (92.16%) | 47 (92.16%) |  |
|  | Yes | 4 (7.84%) | 4 (7.84%) |  |
| Infant Sex | |  |  | p=1 |
|  | Female | 22 (43.14%) | 22 (43.14%) |  |
|  | Male | 29 (56.86%) | 29 (56.86%) |  |
| Ethnicity | |  |  | p=1 |
|  | Asian/Pacific Islander | 2 (3.92%) | 2 (3.92%) |  |
|  | Black | 2 (3.92%) | 2 (3.92%) |  |
|  | Other | 9 (17.65%) | 9 (17.65%) |  |
|  | White | 38 (74.51%) | 38 (74.51%) |  |
| Method of Conception | |  |  | p=1 |
|  | ICSI | 2 (3.92%) | 2 (3.92%) |  |
|  | Ovarian Stimulation only | 1 (1.96%) | 1 (1.96%) |  |
|  | Spontaneous | 46 (90.2%) | 46 (90.2%) |  |
|  | Unknown | 2 (3.92%) | 2 (3.92%) |  |
| Gestational Age (weeks) | | 38.943 (0.676) | 38.992 (0.887) | p= 0.7543 |
| Birth Weight (kg) | | 3.481 (0.685) | 3.676 (0.615) | p= 0.1347 |

**Table S5**. Assessing effect modification of the association between GDM and placenta methylation level estimated by pyrosequencing by maternal age, ethnicity, and pre-pregnancy BMI in our validation cohort (RICHS) and verification set (HEBC).

| **Effect Modifier‡** | | **Interaction with GDM† [Coef (95% CI)]** | | | |
| --- | --- | --- | --- | --- | --- |
| **Maternal Age (years)** | | **Validation Set** | | **Verification Set** | |
|  |  | **Low (range: 22-33; n=75)** | **High (range: 34-40; n=27)** | **Low (range: 20-33; n=37)** | **High (range: 34-43; n=45)** |
|  | *HLA-DOA* | -2.79 (-6.50, 0.92) | -4.04 (-10.30, 2.20) | -8.73* (-15.59, -1.86) | -9.61* (-15.86, -3.36) |
|  | *HLA-H/HLA-J* | -3.56 (-8.86, 1.75) | 3.65 (-5.30, 12.60) | 2.42 (-6.03, 10.87) | 6.82 (-0.88, 14.52) |
|  | *SNRPN/SNURF* | -0.09 (-5.09, 4.90) | -1.21 (-9.64, 7.20) | -4.46 (-13.59, 4.7) | -4.35 (-12.55, 3.8) |
|  | *CCDC181* | -5.81* (-10.89, -0.72) | 7.24 (-1.32, 15.81) | 5.70 (-3.50, 14.87) | 12.41* (4.10, 20.77) |
| **Pre-preg. BMI (kg/m^2^)** | | **Low (range: 15.93- 24.61; n=29)** | **High (range: 25.36- 40.51; n=73)** | **Low (range: 17.92- 24.80; n=39)** | **High (range: 24.96- 39.70; n=43)** |
|  | *HLA-DOA* | 2.35 (-3.55, 8.25) | -5.12* (-8.83, -1.42) | -10.80* (-17.30, -4.30) | -7.82* (-13.99, -1.70) |
|  | *HLA-H/HLA-J* | 2.57 (-5.99, 11.13) | -3.14 (-8.51, 2.24) | 0.20 (-7.90, 8.30) | 8.88* (1.20, 16.57) |
|  | *SNRPN/SNURF* | -0.05 (-8.10, 8.02) | -0.59 (-5.60, 4.47) | -5.08 (-13.78, 3.62) | -3.72 (-12.09, 4.64) |
|  | *CCDC181* | -2.42 (-10.89, 6.06) | -2.41 (-7.73, 2.91) | 4.69 (-3.94, 13.30) | 13.52* (5.33, 21.70) |
| **Ethnicity** | | **White**  **(n=76)** | **Non-White**  **(n=26)** | **White**  **(n=46)** | **Non-White**  **(n=36)** |
|  | *HLA-DOA* | -3.03 (-6.72, 0.65) | -2.92 (-9.23, 3.38) | -11.02* (-16.99, -5.04) | -6.96* (-13.70, -0.22) |
|  | *HLA-H/HLA-J* | -1.77 (-7.09, 3.54) | -0.93 (-10.02, 8.16) | 2.89 (-4.65, 10.44) | 7.27 (-1.24, 15.78) |
|  | *SNRPN/SNURF* | 0.02 (-4.93, 4.97) | -1.78 (-10.25, 6.69) | -4.70 (-12.77, 3.4) | -4.03 (-13.18, 5.1) |
|  | *CCDC181* | -2.70 (-7.90, 2.51) | -1.63 (-10.54, 7.27) | 8.38* (0.36, 16.40) | 10.56* (1.51, 19.60) |

† A linear mixed model was used to identify changes in methylation level associated with GDM, adjusting for maternal age (years), pre-pregnancy BMI (kg/m^2^), infant sex, maternal smoking (yes/no), and self-reported ethnicity, with a random intercept for sample.

‡Possible effect modification by maternal ethnicity (White/Non-White), maternal age (dichotomized by median age in HEBC), and pre-pregnancy BMI (dichotomized by median BMI in HEBC) was assessed among the pyrosequenced regions by a Wald test of the interaction term. Associations are reported separately for each strata.

* p<0.05

**Table S6.** Among maternal blood HEBC samples, associations between GDM and methylation of candidate regions assayed by pyrosequencing.

|  | | **Association with GDM [Coef (95% CI)]** | |
| --- | --- | --- | --- |
| **Gene in proximity** | | **Locus Closest to Candidate on Microarray†** | **Regional change‡** |
|  | *HLA-DOA* | -2.965 (-6.896, 0.967) | -1.764 (-6.009, 2.481) |
|  | *HLA-H/HLA-J* | -0.042 (-9.423, 9.339) | 0.476 (-8.632, 9.585) |

†Association between GDM and methylation of CpG site assayed by pyrosequencing in closest proximity to the site chosen for validation based on methylation array data. Linear model adjusted for maternal age (years), pre-pregnancy BMI (kg/m^2^), infant sex, maternal smoking (yes/no), and self-reported ethnicity.

‡Change in methylation associated with GDM across pyrosequenced loci modeled using linear mixed models with a random intercept for sample, adjusting for the same covariates.

**Table S7**. Assessing effect modification of the association between GDM and maternal blood methylation level estimated by pyrosequencing by maternal age, ethnicity, and pre-pregnancy BMI in HEBC.

| **Effect Modifier‡** | | **Interaction with GDM† [Coef (95% CI)]** | |
| --- | --- | --- | --- |
| **Maternal Age (years)** | | **Verification Set** | |
|  |  | **Low (range: 20-33; n=37)** | **High (range: 34-43; n=45)** |
|  | *HLA-DOA* | 0.37 (-6.30, 7.05) | -2.76 (-8.50, 2.98) |
|  | *HLA-H/HLA-J* | 5.29 (-9.1, 19.63) | -2.66 (-14.8, 9.52) |
| **Pre-preg. BMI (kg/m^2^)** | | **Low (range: 17.92- 24.80; n=39)** | **High (range: 24.96- 39.70; n=43)** |
|  | *HLA-DOA* | 3.22 (-2.93, 9.38) | -6.04* (-11.72, -0.36) |
|  | *HLA-H/HLA-J* | -3.44 (-17.1, 10.20) | 4.11 (-8.70, 16.90) |
| **Ethnicity** | | **White**  **(n=46)** | **Non-White**  **(n=36)** |
|  | *HLA-DOA* | 0.82 (-4.69, 6.34) | -5.65 (-12.45, 1.14) |
|  | *HLA-H/HLA-J* | 3.05 (-8.83, 14.93) | -3.50 (-18.34, 11.35) |

† A linear mixed model was used to identify changes in methylation level associated with GDM, adjusting for maternal age (years), pre-pregnancy BMI (kg/m^2^), infant sex, maternal smoking (yes/no), and self-reported ethnicity, with a random intercept for sample.

‡Possible effect modification by maternal ethnicity (White/Non-White), maternal age (dichotomized by median age in HEBC), and pre-pregnancy BMI (dichotomized by median BMI in HEBC) was assessed among the pyrosequenced regions by a Wald test of the interaction term. Associations are reported separately for each strata.

* p<0.05

**Table S8.** Most significant changes in gene-level expression associated with GDM in adjusted model; restricting to top 20 unique coding RefSeq genes.

| Gene ID | Coefficient† | P-value‡ | Position | Strand | Probes |
| --- | --- | --- | --- | --- | --- |
| *HLA-C* | -0.252 | 0.000101 | chr6:31236526-31239913 | - | 181 |
| *HLA-C* | -0.253 | 0.000118 | chr6_cox_hap2:2749781-2753168 | - | 171 |
| *MIR4534* | 0.172 | 0.000187 | chr22:38384801-38384860 | + | 30 |
| *HLA-DQA2* | -0.271 | 0.00021 | chr6_qbl_hap6:3941202-3947061 | + | 66 |
| *HLA-DQA2* | -0.300 | 0.00021 | chr6_mann_hap4:4166277-4171838 | + | 60 |
| *CT47A7* | 0.102 | 0.000373 | chrX:120087137-120090454 | - | 30 |
| *ZNF695* | 0.047 | 0.000403 | chr1:247108849-247171395 | - | 116 |
| *GPR174* | -0.145 | 0.000591 | chrX:78426469-78427726 | + | 30 |
| *HLA-DQA2* | -0.288 | 0.000664 | chr6_cox_hap2:4155020-4161095 | + | 60 |
| *HLA-DQA2* | -0.256 | 0.000695 | chr6_ssto_hap7:4140787-4146657 | + | 67 |
| *PELO* | -0.096 | 0.000724 | chr5:52083774-52121044 | + | 100 |
| *NLGN4Y-AS1* | 0.086 | 0.000774 | chrY:16905522-16915913 | - | 35 |
| *MIR4653* | -0.131 | 0.000884 | chr7:100802754-100802836 | + | 30 |
| *HLA-DQA2* | -0.296 | 0.000888 | chr6_apd_hap1:3997436-4003267 | + | 58 |
| *HS6ST3* | -0.102 | 0.00089 | chr13:96743093-97491816 | + | 40 |
| *HLA-DQA2* | -0.240 | 0.000893 | chr6:32709119-32714992 | + | 70 |
| *HLA-DQA2* | -0.233 | 0.001021 | chr6_mcf_hap5:4046301-4052169 | + | 68 |
| *MIR30B* | -0.109 | 0.001022 | chr8:135812763-135812850 | - | 30 |
| *CD96* | -0.069 | 0.001029 | chr3:111260926-111384597 | + | 248 |
| *SAMD9L* | -0.258 | 0.001186 | chr7:92759368-92777682 | - | 180 |
| *HLA-B* | -0.258 | 0.001357 | chr6_cox_hap2:2834338-2837710 | - | 173 |
| *SLC16A6* | -0.070 | 0.00146 | chr17:66263167-66287405 | - | 80 |
| *MBLAC2* | -0.077 | 0.001484 | chr5:89754020-89770585 | - | 80 |
| *FGL2* | -0.351 | 0.001487 | chr7:76822688-76829150 | - | 30 |
| *LOC643406* | 0.093 | 0.001499 | chr20:5451842-5457780 | + | 50 |
| *CLEC4E* | -0.145 | 0.001526 | chr12:8685901-8693559 | - | 90 |
| *HLA-B* | -0.244 | 0.001566 | chr6_ssto_hap7:2655408-2658765 | - | 213 |
| *HLA-A* | -0.164 | 0.001594 | chr6:29909037-29913661 | + | 230 |

**†**Adjusting for chart-abstracted maternal age (years), pre-pregnancy BMI (kg/m^2^), infant sex, maternal smoking (yes/no), and independent surrogate variables associated with putative sources of internal bias measured with error; specifically, self-reported maternal ethnicity and our indicators for batch

**‡** Robust standard errors used to account for possible heteroscedasticity

**Table S9.** Spearman correlation between methylation of loci chosen for validation and expression of genes in MHC region (chr6: 28477797-33448354); restricting to pairwise comparisons with p<0.05.

| Comparison | | Spearman (ρ) | P-value | q-value (FDR) |
| --- | --- | --- | --- | --- |
| cg23681866 (*HLA-H/HLA-J*) | | | | |
|  | *PSORS1C2* | 0.313 | 0.010 | 0.389 |
|  | *LOC401242* | 0.308 | 0.011 | 0.392 |
|  | *LOC100129636* | 0.290 | 0.016 | 0.402 |
|  | *PPP1R11* | 0.257 | 0.029 | 0.420 |
|  | *OR2H1* | 0.246 | 0.035 | 0.425 |
|  | *STK19* | 0.244 | 0.036 | 0.425 |
| cg08147094 (*HLA-DOA*) | | | | |
|  | *HLA-C* | 0.475 | 0.000 | 0.287 |
|  | *HLA-F* | 0.396 | 0.001 | 0.340 |
|  | *HLA-A* | 0.364 | 0.003 | 0.360 |
|  | *HLA-L* | 0.346 | 0.005 | 0.369 |
|  | *HLA-B* | 0.332 | 0.007 | 0.379 |
|  | *HLA-DQA2* | 0.323 | 0.009 | 0.384 |
|  | *OR12D2* | 0.306 | 0.011 | 0.391 |
|  | *HLA-H* | 0.295 | 0.015 | 0.401 |
|  | *HLA-DOA* | 0.294 | 0.015 | 0.401 |
|  | *HLA-E* | 0.271 | 0.024 | 0.413 |
|  | *TAP1* | 0.269 | 0.024 | 0.413 |
|  | *TRIM27* | 0.246 | 0.035 | 0.425 |
|  | *MIR219-1* | 0.232 | 0.044 | 0.431 |
|  | *HLA-DPB1* | 0.231 | 0.047 | 0.433 |
|  | *HLA-G* | 0.229 | 0.048 | 0.434 |
|  | *HLA-DQA1* | 0.225 | 0.050 | 0.435 |

**Table S10.** Association between GDM and expression of genes estimated by qRT-PCR in adjusted models in our validation cohort (RICHS) and verification set (HEBC).

| Gene | Association with GDM† [Coef (95% CI)] | | |
| --- | --- | --- | --- |
|  | Validation Set (n=102) | | Verification Set (n=81) |
| *HLA-C* | 0.149 (-0.258, 0.557) | -0.711 (-1.522, 0.100) | |
| *HLA-B* | 0.042 (-0.347 , 0.431) | -0.410 (-1.138, 0.318) | |
| *GPR174* | 0.458 (-0.579 , 1.495) | 0.291 (-0.643 , 1.225) | |

†Expression estimated by qRT-PCR was modeled as a function of GDM, adjusting for maternal age (years), pre-pregnancy BMI (kg/m^2^), infant sex, maternal smoking (yes/no), and self-reported ethnicity, using robust standard errors.

**Table S11.** Biological pathway enrichment among genes in proximity to methylation changes associated with GDM. Gene ontology (GO) enrichment was assessed among the genes in proximity to the 648 CpG loci associated with GDM in our adjusted models at α-level=0.001. Restricted to ontologies with p<0.05.

| GO Term (Biological Process) | Size of GO | Number of Subset in GO | OR | P-value | Q-value |
| --- | --- | --- | --- | --- | --- |
| respiratory burst | 25 | 4 | 10.15769 | 9.00E-04 | 0.2337 |
| organic hydroxy compound catabolic process | 61 | 6 | 4.273931 | 0.0032 | 0.2337 |
| multi-organism metabolic process | 104 | 7 | 2.933183 | 0.0033 | 0.2337 |
| cellular component disassembly | 429 | 23 | 2.253664 | 0.0035 | 0.2337 |
| response to xenobiotic stimulus | 158 | 9 | 2.413078 | 0.0041 | 0.2337 |
| one-carbon metabolic process | 32 | 4 | 5.466162 | 0.0097 | 0.407143 |
| vitamin transport | 23 | 3 | 5.913333 | 0.01 | 0.407143 |
| digestion | 112 | 7 | 2.738941 | 0.014 | 0.49875 |
| ketone biosynthetic process | 32 | 4 | 5.466162 | 0.0172 | 0.49875 |
| phagosome maturation | 47 | 4 | 3.838921 | 0.0175 | 0.49875 |
| drug metabolic process | 33 | 3 | 3.940404 | 0.0257 | 0.634125 |
| alditol phosphate metabolic process | 33 | 3 | 3.940404 | 0.0267 | 0.634125 |

**Table S12.** Biological pathway enrichment among genes with differential expression associated with GDM. Gene ontology (GO) enrichment was assessed among the 171 differentially expressed genes associated with GDM in our adjusted models at α-level=0.01. Restricted to ontologies with p<0.05.

| GO Term (Biological Process) | Size of GO | Number of Subset in GO | OR | P-value | Q-value |
| --- | --- | --- | --- | --- | --- |
| immune response | 1358 | 31 | 4.059239 | 1.54E-09 | 2.80E-07 |
| cell killing | 94 | 6 | 10.61429 | 3.83E-05 | 0.003484 |
| response to cytokine | 601 | 14 | 3.74746 | 6.02E-05 | 0.003654 |
| defense response | 1454 | 22 | 2.492199 | 0.000313 | 0.012644 |
| response to biotic stimulus | 714 | 14 | 3.136725 | 0.000347 | 0.012644 |
| antigen processing and presentation | 230 | 7 | 4.697704 | 0.001086 | 0.032954 |
| positive regulation of response to stimulus | 1498 | 21 | 2.256534 | 0.001499 | 0.038984 |
| defense response to other organism | 379 | 8 | 3.304325 | 0.004182 | 0.095132 |
| detection of external biotic stimulus | 23 | 2 | 15.96006 | 0.008493 | 0.162448 |
| behavior | 608 | 10 | 2.534152 | 0.008926 | 0.162448 |
| positive regulation of RNA biosynthetic process | 1199 | 2 | 0.238092 | 0.026773 | 0.442976 |
| response to drug | 378 | 6 | 2.447308 | 0.042155 | 0.639349 |
| positive regulation of biosynthetic process | 1477 | 4 | 0.386798 | 0.048133 | 0.673861 |

**Table S13.** Pyrosequencing Assay Information.

| **CpG from 450K** | **Gene Symbol** | **Epigendx Assay ID** | **From ATG** | **From TSS** | **Chromosome Location** | **# of CpG in Assay** | **CpGs Analyzed** | **# of SNPs** | **Assay includes CpG from 450K** | **CpG # matches 450K** |
| --- | --- | --- | --- | --- | --- | --- | --- | --- | --- | --- |
| cg25464921 | *CCDC181* | ADS3941-FS | -35566 to -35482 | +177 to +261 | chr1:169429731-169429639 | 10 | #1-8 | 2 | Yes | CpG #6 |
| cg08147094 | *HLA-DOA* | ADS1386-FS3 | -362 to  -396 | -286 to -320 | Chr6: 32977675-32977709 | 2 | #1-2 | - | No* | CpG #1 is closest proximity |
| cg23681866 | *HLA-H/ HLA-J* | ADS3938-FS1 | +936 to +992 | +936 to +992 | chr6:29895171-29895227 | 6 | #1-2 | - | Yes | CpG #2 |
| cg18506672 | *SNRPN* | ADS3945  -FS1 | -19348 to -19291 | +131460 to +131517 | Chr15:25200253-  25200310 | 2 | #1-2 | 3 | Yes | CpG #1 |

*For HLA-DOA assay analyzes the subsequent 2 CpG sites following site cg08147094.

**Table S14.** qRT-PCR Primer Information.

| IDT Assay ID | Gene Symbol | Exon Location | Transcripts Hits |
| --- | --- | --- | --- |
| Hs.PT.39a.22214836 | *GAPDH** | 2 - 3 | NM_002046 |
| Hs.PT.58.38856671.g | *HLA-B* | 8 - 8 | NM_005514 |
| Hs.PT.58.22217602 | *HLA-C* | 5 - 8 | NM_001243042; NM_002117 |
| Hs.PT.58.28349515.g | *GPR174* | 1 - 1 | NM_032553 |

*Housekeeping gene used for normalization
