# Supplementary material for: Epigenome-wide and transcriptome-wide analyses reveal gestational diabetes is associated with alterations in the human leukocyte antigen complex
Source: Clin Epigenetics. 2015 Aug 5;7(1):79. doi: 10.1186/s13148-015-0116-y (PMC4524439; doi:10.1186/s13148-015-0116-y)
Supplement: Additional file 2: Figure S1. — Distribution of maternal characteristics in HEBC and validation cohort (RICHS). Figure S2. Plotting the first two principle components for methylation and expression in the MHC region (chr6: 28477797–33448354) for GDM cases (blue) and matched controls (red). Figure S3. The subset of 20 genes driving the first two principle component loadings for expression (|rotation| > 0.1) in the MHC region (chr6: 28477797–33448354) for GDM cases (blue) and matched controls (red). [file 13148_2015_116_MOESM2_ESM.pdf]

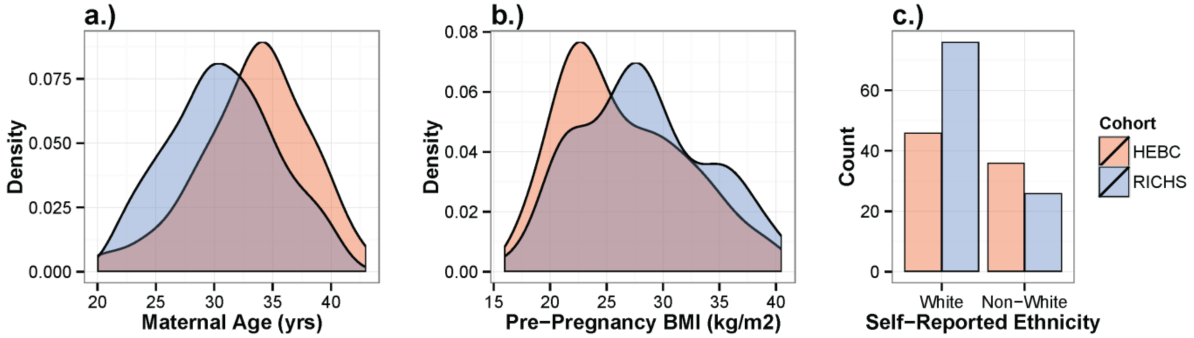

Supplemental Figure 1. Distribution of maternal characteristics in HEBC and validation cohort (RICHS).

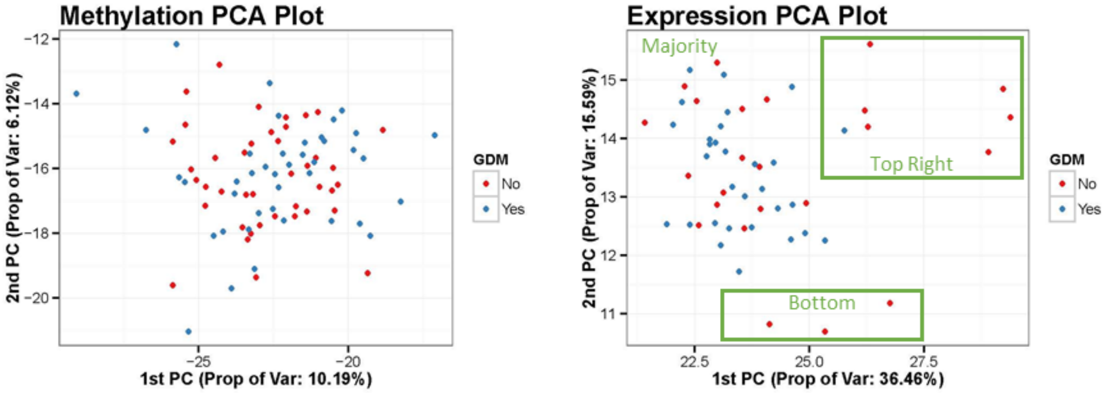

Supplemental Figure 2. Plotting the first two principle components for methylation and expression in the MHC region (chr6: 28477797-33448354) for GDM cases (blue) and matched controls (red). While there are no clear groupings by methylation, there appears to be clustering in the expression plot, categorized as: the majority, the top right, and bottom. Between these three groups, there is no significant difference in maternal age, pre-pregnancy BMI, ethnicity, birth weight or gestational age. The two side clusters are more likely to have GDM ($p<0.05$). The subset of 20 genes driving the first two principle component loadings for expression ($|rotation|>0.1$) represent genes down-regulated with GDM in Figure 2 (additional information: Supplemental Figure 3).

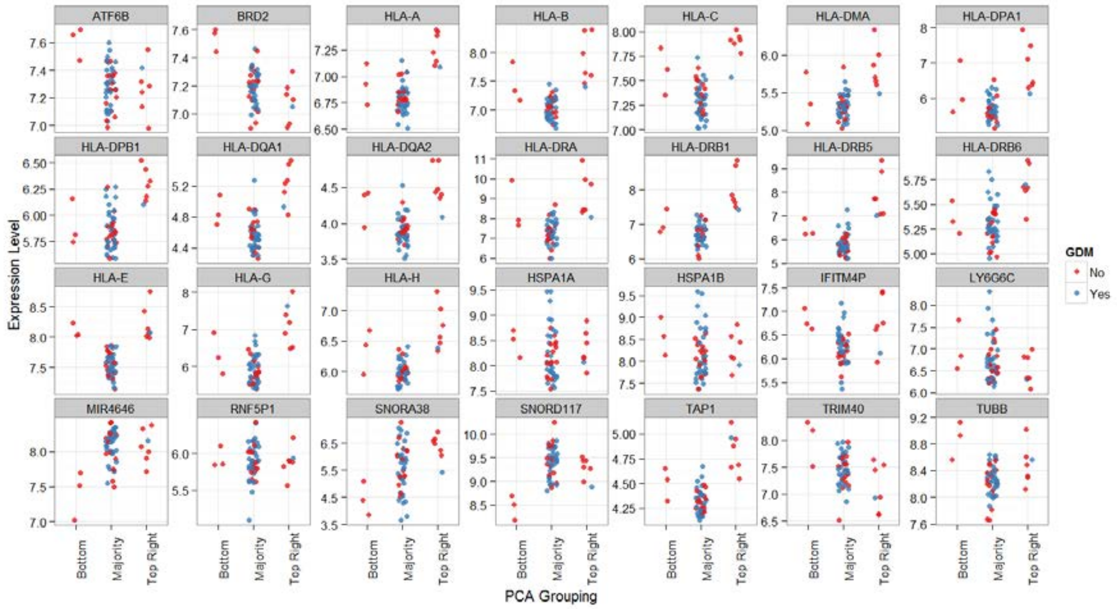

Supplemental Figure 3. The subset of 20 genes driving the first two principle component loadings for expression (|rotation|>0.1) in the MHC region (chr6: 28477797-33448354) for GDM cases (blue) and matched controls (red). Individuals separated by groupings observed in Supplemental Figure 2.
